# Tuning Schottky Barrier of Single-Layer MoS_2_ Field-Effect Transistors with Graphene Electrodes

**DOI:** 10.3390/nano12173038

**Published:** 2022-09-01

**Authors:** A-Rang Jang

**Affiliations:** Division of Electrical, Electronic and Control Engineering, Kongju National University, Cheonan 31080, Korea; arjang@kongju.ac.kr

**Keywords:** graphene, molybdenum disulfide, Schottky barrier

## Abstract

Two–dimensional materials have the potential to be applied in flexible and transparent electronics. In this study, single-layer MoS_2_ field-effect transistors (FETs) with Au/Ti–graphene heteroelectrodes were fabricated to examine the effect of the electrodes on the electrical properties of the MoS_2_ FETs. The contact barrier potential was tuned using an electric field. Asymmetrical gate behavior was observed owing to the difference between the MoS_2_ FETs, specifically between the MoS_2_ FETs with Au/Ti electrodes and those with graphene electrodes. The contact barrier of the MoS_2_ FETs with Au/Ti electrodes did not change with the electric field. However, the contact barrier at the MoS_2_–graphene interface could be modulated. The MoS_2_ FETs with Au/Ti–graphene electrodes exhibited enhanced on/off ratios (~10^2^ times) and electron mobility (~2.5 times) compared to the MoS_2_ FETs with Au/Ti electrodes. These results could improve the understanding of desirable contact formation for high-performance MoS_2_ FETs and provide a facile route for viable electronic applications.

## 1. Introduction

Two–dimensional (2D) materials have attracted significant attention as potential candidates for next-generation electronics [1,2]. Graphene is considered to be one of the most promising 2D materials because of its unique electrical, mechanical, and optical properties. However, the widespread use of graphene in viable electronic device applications is limited by its zero-bandgap property, which considerably decreases the on/off ratio [3,4,5,6]. To overcome this limitation, graphene nanoribbons [7,8], bilayer graphene [9,10,11,12], and modified device architectures, such as vertical tunneling transistors, have been developed [13,14]. Although these devices have improved the on/off ratio, other desirable properties, such as mobility and current density, have deteriorated. Thus, there is an urgent requirement for 2D materials, including transition metal dichalcogenides (TMDs), with an appropriate bandgap and reasonable mobility to replace graphene. MoS_2_ is one of the most promising TMDs because its bandgap is 1.3–1.8 eV depending on the number of layers. Single-layer MoS_2_ films have a direct bandgap of 1.8 eV, whereas multilayer MoS_2_ films have an indirect bandgap of 1.2 eV [15,16,17]. Owing to these unique properties, MoS_2_ has been intensively studied for electronic and optoelectronic applications. In recent years, it has become possible to synthesize large-area single-layer MoS_2_ via chemical vapor deposition (CVD) [18,19,20,21,22]. This has provided a major opportunity for next-generation electronic device applications. However, the contact barrier issue must be studied for electronic device applications of 2D materials [23,24]. Moreover, the performance of MoS_2_ field-effect transistors (FETs) is lower than the theoretically predicted performance [25]. This discrepancy has been explained on the basis of charged impurities and localized states in MoS_2_ [26,27,28]. Dominant scattering processes decrease carrier mobility. In addition, the contact at MoS_2_–metal electrode interfaces is a critical issue. A tunneling barrier that is formed at the interface of a metal contact in an MoS_2_ device [29] significantly reduces carrier mobility in single–layer MoS_2_. This is one of the main reasons for the poor performance of single-layer MoS_2_ FETs. Sulfur atoms mediate the hybridization between a contact metal and Mo atoms, resulting in the tuning of the bandgap [30]. Furthermore, the bandgap of single-layer MoS_2_ can be determined by the strength of the Mo–S covalent bonding [31]. Therefore, a systematic study of the effects of electrode materials on the performance of MoS_2_ FETs can help resolve this critical issue and find a reliable method of improving the electrical properties of MoS_2_ FETs. A charge accumulation region forms at metal–MoS_2_ interfaces when a metal contact is used. This generally leads to the formation of an interface electric dipole, which modifies the interface band alignment [30]. This results in poor contact and an unexpected contact barrier between the metal and MoS_2_. Owing to the challenges associated with metal electrodes, graphene has been considered as a suitable electrode material for MoS_2_ FETs. Graphene and single-layer MoS_2_ bond via van der Waals (vdW) forces, thereby creating a pristine interface. Furthermore, the contact barrier between graphene and MoS_2_ can be controlled by tuning the work function of graphene (4.5 eV), which is quite similar to that of MoS_2_ [32]. As the work function of graphene can be readily tuned by applying an electric field, graphene-based heterostructures have recently been studied in electronic devices [33,34,35,36]. For instance, the Schottky barrier formed between graphene and silicon can be tuned by approximately 200 meV as a function of the gate voltage [13]. Therefore, the contact barrier between graphene and MoS_2_ can be tuned by applying an electric field.

Herein, we report high-performance single–layer MoS_2_ FETs with graphene electrodes that exhibit a considerable enhancement in the on/off ratio (~10^2^ times) and electron mobility (~2.5 times) compared to the MoS_2_ FETs with Au/Ti electrodes. We show that the contact barrier potential of the MoS_2_ FETs with graphene electrodes can be effectively tuned by applying an electric field. The work function of graphene becomes higher than that of MoS_2_ at a negative bias voltage, resulting in the formation of a Schottky barrier. Similarly, the work function of graphene becomes lower than that of MoS_2_ at a positive bias voltage, resulting in the formation of an ohmic barrier. The contact barrier between MoS_2_ and graphene can be easily tuned using graphene electrodes. Thus, the on/off ratio and electron mobility of the MoS_2_ FET can be improved by tuning the contact barrier.

## 2. Materials and Methods

### 2.1. Graphene Growth and Transfer

Graphene was synthesized on a copper foil (99.8% purity, 0.025 mm thick, Alfa Aesar, Haverhill, MA, USA) using CVD at a growth temperature of 1050 °C with 10 sccm of H_2_ and 15 sccm of CH_4_ [37]. Then, the full side of the foil that faced upwards during synthesis was covered with poly(methyl methacrylate) (PMMA) (AR–N 7500.18, Allresist, Strausberg, Germany) via spin coating (4000 rpm for 60 s). The remaining graphene on the Cu foil that faced downwards during the synthesis was removed using O_2_ plasma (Femto, Diener, Ebhausen, Germany). The Cu foil was completely etched using 0.1 M ammonia persulfate (Sigma Aldrich, St. Louis, MO, USA). The PMMA/graphene layer was washed several times with fresh deionized water. Finally, the PMMA/graphene layer floated on the surface of the water, and it was transferred to a SiO_2_ substrate. The transferred PMMA/graphene layer was patterned using electron beam lithography (Nanobeam nB4, NBL, Cambridge, UK) as shown in Figure S1a.

### 2.2. Fabrication of the MoS_2_ Field-Effect Transistor

**Figure 1:**
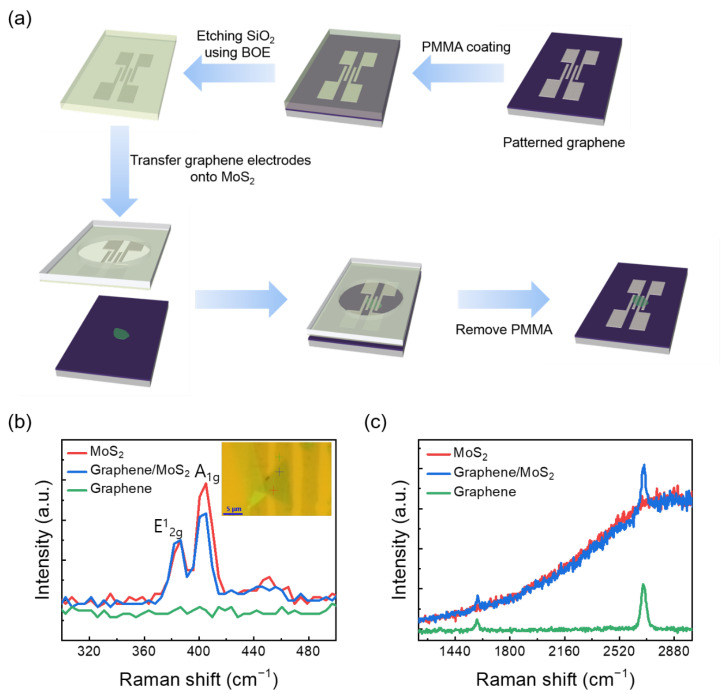
(**a**) Schematics illustration of the fabrication process for MoS_2_ FET with graphene electrode. Raman spectroscopy of mechanically exfoliated single–layer MoS_2_ (red), chemical vapor deposition (CVD)-grown graphene on single-layer MoS_2_ (blue), and CVD-grown graphene (green). (**b**) MoS_2_ region, and (**c**) graphene region of the Raman spectrum (the insert of (**b**) shows the Raman analysis position by cross mark).

As shown in Figure S1b, single-layer MoS_2_ was prepared via mechanical exfoliation from a bulk MoS_2_ flake (429ML–AB, SPI Supplies, West Chester, PA, USA). To fabricate MoS_2_ FET with graphene electrode, a dry transfer process was employed [38]. Patterned graphene was transferred onto single–layer MoS_2_ flake after the alignment position using a micromanipulator (NMO–203, Narishige, Tokyo, Japan) (Figure 1a). Au/Ti electrodes were patterned using electron beam lithography with a positive electron beam resist (AR–P 671.04, Allresist, Strausberg, Germany). This was followed by metal deposition (Ti (5 nm)/Au (45 nm)) and a lift-off process.

### 2.3. Characterization of the MoS_2_ Thin Film and Field–Effect Transistor

Mechanical exfoliation was employed to extract high-quality single-layer MoS_2_ from bulk MoS_2_ [3]. Then, single–layer MoS_2_ was transferred onto a silicon wafer with a 300 nm thick SiO_2_ layer. Raman spectroscopy (Alpha 300R, WiTec, Ulm, Germany) was used to determine the number of layers of MoS_2_ [39]. The Raman spectrum of MoS_2_ revealed a peak spacing of less than 20 cm^−1^ between the E_2g_ and A_1g_ modes, indicating that single-layer MoS_2_ was formed. A 532 nm laser with a power of 1 mW was used as an excitation source. The exposure time was 1 s, and calibration was performed using a reference Si peak position of 520 cm^−1^. The fabricated MoS_2_ FETs were loaded into a vacuum chamber (Lake Shore) for electrical measurements. The electrical properties of the MoS_2_ FETs with graphene–graphene electrodes, Au/Ti–Au/Ti electrodes, and graphene–Au/Ti electrodes were characterized in vacuum (under 10^−4^ Torr) using a semiconductor parameter analyzer (4200-SCS with a preamplifier unit, Keithley, Cleveland, OH, USA) for comparison.

## 3. Results and Discussion

**Figure 2:**
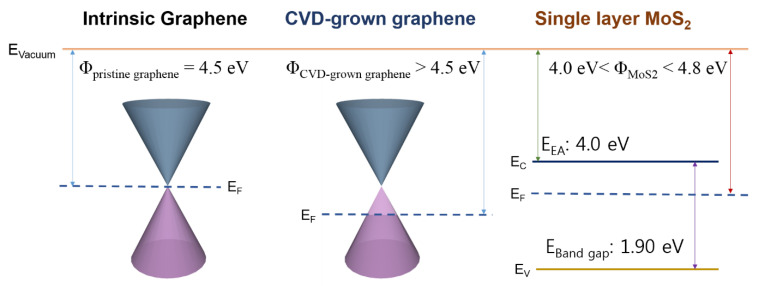
Schematic band diagram of intrinsic graphene, CVD-grown graphene, and single-layer MoS_2_.

**Figure 3:**
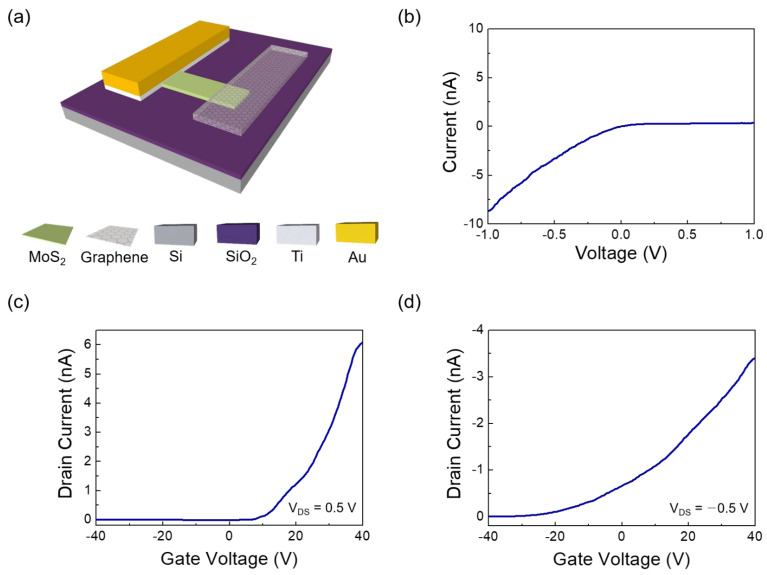
Schematic and electrical properties of MoS_2_ field-effect transistor (FET) with heteroelectrodes. (**a**) Schematic of MoS_2_ FET with heteroelectrodes; (**b**) I_DS_–V_DS_ output characteristics; (**c**) I_DS_–V_g_ transfer characteristics at V_DS_ = 0.5 V; (**d**) I_DS_–V_g_ transfer characteristics at V_DS_ =−0.5 V.

**Figure 4:**
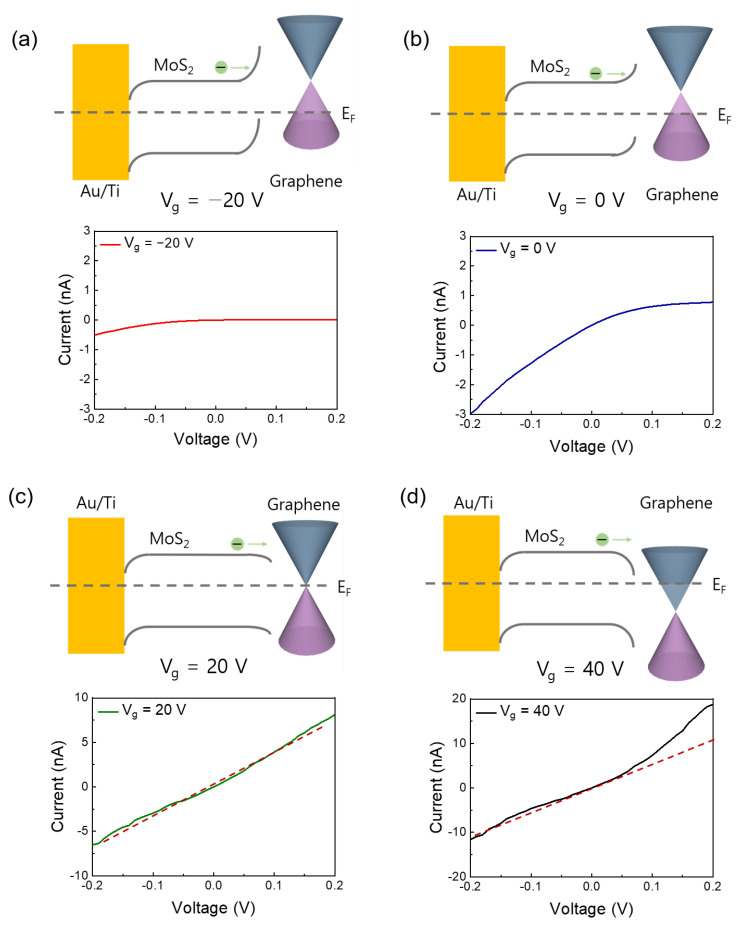
Band diagrams and electrical properties of the MoS_2_ FET with Au–graphene heteroelectrode at different gate voltages ((**a**) −20 V, (**b**) 0 V, (**c**) 20 V, and (**d**) 40 V).

**Figure 5:**
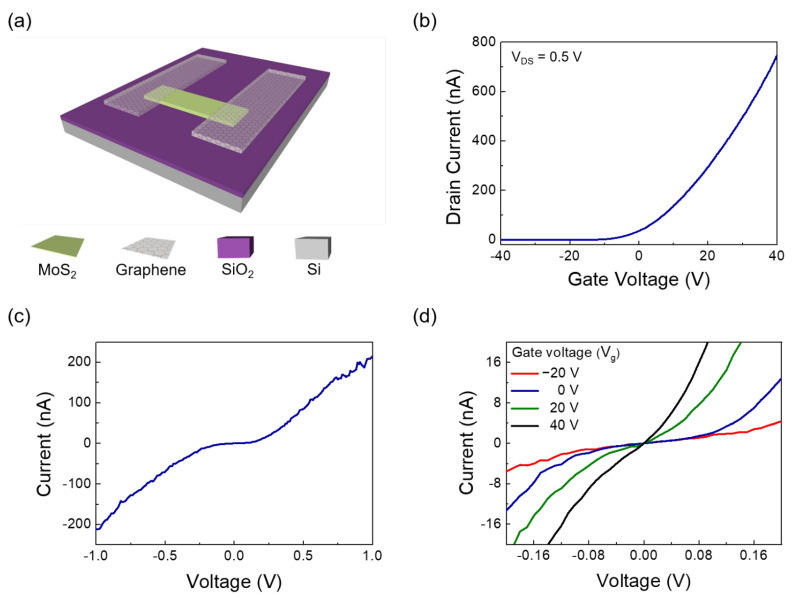
Schematic and electrical properties of MoS_2_ FET with graphene electrodes. (**a**) Schematic of MoS_2_ FET with graphene electrodes; (**b**) I_DS_–V_g_ transfer characteristics at V_DS_ = 0.5 V; (**c**) I_DS_–V_DS_ output characteristics; (**d**) I_DS_–V_DS_ characteristics at different gate bias voltages.

**Figure 6:**
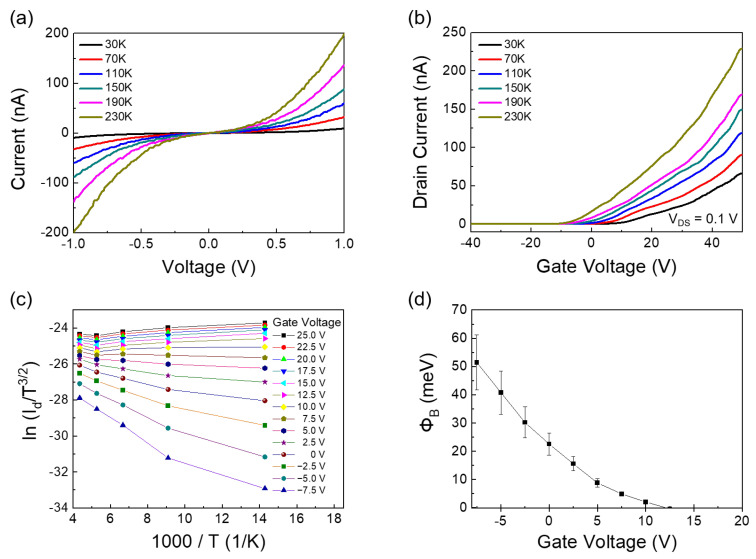
Temperature-dependent electrical transport of the MoS_2_ FET with graphene electrode. (**a**) Current voltage characteristics and (**b**) I_DS_–V_g_ transfer characteristics from 30 K to 230 K, for source-drain bias voltage of 0.1 V. (**c**) Linear fit of the Arrhenius plot, $\ln\left(I_{d}/T^{3/2}\right)\;\mathrm{vs}.\;$1000/*T* as function of V_g_. (**d**) The Schottky barrier of MoS_2_ FET with graphene electrode depends on the gate voltage.

Figure 1b,c show the Raman spectra of single-layer MoS_2_, graphene on single-layer MoS_2_, and graphene, respectively. The MoS_2_ and graphene/MoS_2_ layers exhibit typical single-layer Raman active modes (~18.27 cm^−1^ of frequency difference between E_2g_ and A_1g_), and the 2D/G ratio of graphene is about 4.06. Therefore, it can be noted that exfoliated MoS_2_ flake has a formation of single layer. The Dirac point of intrinsic graphene is at zero gate voltage, the work function of which is approximately 4.5 eV [32]. As shown in Figure S2, the Dirac point of the CVD-grown graphene electrode was measured at 22.5 V, owing to the hole doping originated from both coupling with dielectric layer of SiO_2_ and exposure to oxygen and moisture [40]. The schematic of the band structure of graphene and MoS_2_ is shown in Figure 2. Graphene and single-layer MoS_2_ were bonded via weak vdW forces. However, MoS_2_ and the metal interfaces formed covalent interactions, causing a change in the electronic structure [30]. This led to unexpected contact resistance. Three different types of single-layer MoS_2_ FETs were fabricated to investigate the effects of the graphene electrode. The first was a single-layer MoS_2_ FET with a Au/Ti–graphene heteroelectrode, as shown in Figure 3a. Highly boron-doped Si (resistance of 0.001 Ω) with a 300 nm thick SiO_2_ layer was used as the substrate. The channel length and width of the mechanically exfoliated MoS_2_ used in the single-layer MoS_2_ FET were ~2 μm and ~4 μm, respectively. Figure 3b shows the asymmetric I_DS_–V_DS_ output characteristics of the single-layer MoS_2_ FET with the Au/Ti–graphene heteroelectrode without the gate voltage. Different contact barriers were generated according to the contact material. An ohmic contact was formed between single-layer MoS_2_ and Au/Ti. A Schottky contact was formed between single-layer MoS_2_ and graphene. Figure 3c shows the I_DS_–V_g_ transfer characteristics for a positive source–drain voltage (V_DS_). The on/off ratio and electron mobility (graphene in the heteroelectrode) were >10^5^ and ~3.2 cm^2^/V∙s, respectively. Figure 3d shows the I_DS_–V_g_ transfer characteristics for a negative drain voltage. The on/off ratio and electron mobility (Au/Ti in the heteroelectrode) were >10^2^ and ~1.2 cm^2^/V∙s, respectively. These results indicated that graphene could be used as an ideal electrode in a single-layer MoS_2_ FET. Mobility was calculated using the following equation: $\mu_{e}=g_{m}\times L/\;C_{g}\times V_{D}\times W;$ where $g_{m}$ is the transconductance, $V_{D}$ is the source–drain voltage, L is the channel length, W is the channel width, and $\;C_{g}$ is the capacitance of 300 nm thick SiO_2_. The MoS_2_ FET with the Au/Ti electrodes exhibited ohmic contact behavior, whereas the MoS_2_ FET with the graphene electrodes exhibited Schottky contact behavior. Multilayer MoS_2_ FETs with exfoliated graphene electrodes also showed ohmic contact behavior [41]. The work function of graphene was approximately 4.5 eV because mechanically exfoliated graphene was almost pure with no doping. Therefore, the single-layer MoS_2_ FET with the graphene electrodes exhibited a Schottky barrier without a gate bias voltage. However, the work function of graphene was electrostatically adjusted to approximately 300 meV for single-layer graphene by tuning the Fermi level (E_F_) by changing the gate voltage by 50 V [32]. The work function of graphene decreased at a positive gate bias voltage. Figure 4 shows the I_DS_–V_DS_ characteristics of the single-layer MoS_2_ FET as a function of the back-gate voltage. The Schottky barrier between graphene and single-layer MoS_2_ was enhanced at a negative gate voltage; thus, current could not flow in the negative gate voltage direction (Figure 4a). As the gate was positively biased, the Schottky barrier between graphene and single-layer MoS_2_ decreased, and the contact barrier between single-layer MoS_2_ and Au/Ti did not change. The I_DS_–V_DS_ output characteristics of the single-layer MoS_2_ FET with the Au/Ti–graphene heteroelectrode (green solid line) showed almost similar with linear (red dashed line) at a gate voltage of 20 V because the work function of graphene became similar to that of single-layer MoS_2_ (Figure 4c). As the gate voltage exceeded 20 V, the current level (black solid line) of the graphene electrode became higher than that of the Au/Ti electrode (Figure 4d). These results showed that the electrical properties of the single-layer MoS_2_ FET were enhanced using the graphene electrodes. A Schottky barrier was formed at the interface of graphene and MoS_2_ in the current-off region; thus, there was no leakage current. However, an ohmic barrier was formed at the interface between graphene and MoS_2_ in the current-on region. Therefore, the on/off ratio and electron mobility of single-layer MoS_2_ were high. The on/off ratio and electron mobility of single–layer MoS_2_ were compared with those of homogeneous electrodes. A single-layer MoS_2_ FET with the graphene electrodes was fabricated, and its electrical properties were measured. Figure 5a shows the schematic of the single-layer MoS_2_ FET with the graphene electrodes, and Figure 5b shows its I_DS_–V_g_ transfer characteristics. The I_DS_–V_DS_ output characteristics shown in Figure 5c confirmed that a Schottky barrier was formed. When an increasingly positive back-gate bias was applied to the single-layer MoS_2_ FET with the graphene electrodes, the Schottky barrier was slightly modified into a clear ohmic contact, as shown in Figure 5d. The on/off ratio and electron mobility were >10^5^ and ~2.3 cm^2^/V∙s, respectively. A single-layer MoS_2_ FET with the Au/Ti electrodes was fabricated, and its electrical properties were measured for comparison. Figure S2a shows the schematic of the single-layer MoS_2_ FET with the Au/Ti electrodes, and Figure S2b shows its I_DS_–V_g_ transfer characteristics. The on/off ratio and electron mobility were >10^3^ and ~0.9 cm^2^/V∙s, respectively. The on/off ratio and electron mobility of the single-layer MoS_2_ FET with the graphene electrodes were ~10^2^ and ~2.5 times higher than those of the single-layer MoS_2_ FET with the Au/Ti electrodes, respectively. To study the barrier height of the MoS_2_ FET with graphene electrode, current voltage characteristics (Figure 6a) and I_DS_–V_g_ transfer characteristics (Figure 6b) were measured at different temperatures. The 2D thermionic emission equation was used to describe the electrical transport behavior of Schottky contacted MoS_2_ devices [41,42].

(1) $$I_{DS}=AA_{2D}^{*}T^{3/2}exp[\frac{q}{k_{B}T}(\Phi_{B}-\frac{V_{DS}}{n})]$$

where A is the contact area of the junction, $A_{2D}^{*}$ is the two–dimensional equivalent Richardson constant, q is the magnitude of the electron charge, $\Phi_{B}$ is the Schottky barrier height, $k_{B}$ is the Boltzmann constant, n is the ideality factor, and $V_{DS}$ is the drain-source bias. Instead of the typical Arrehenius plot, $\ln\left(I_{d}/T^{2}\right)$ versus 1000/*T* for three-dimensional semiconductors, $\ln\left(I_{d}/T^{3/2}\right)$ versus 1000/*T* was used because here the semiconducting channel is two-dimensional. The $\ln\left(I_{d}/T^{3/2}\right)$ versus 1000/*T* of MoS_2_ FET with graphene electrodes for various values of $V_{g}$ is shown in Figure 6c. Based on Equation (1), the height of the Schottky barrier can be deduced as Equation (2):

(2) $$y_{intercept}=-\frac{q}{1000k_{B}}\Phi_{B}$$

In the MoS_2_ FET with graphene electrodes, the Schottky barrier is decreased dramatically—from 51.5 meV to 0 meV—with the back gate voltage changing from −7.5 to 12.5 V, as shown in Figure 6d. The change of the Schottky barrier in the MoS_2_ FET with graphene electrodes comes from changes in work function of graphene.

## 4. Conclusions

This work demonstrates the enhancement of the electrical properties of an MoS_2_ FET with graphene electrodes by tuning the contact barrier using an electric field. The MoS_2_ FET with a Au/Ti–graphene heteroelectrode shows a clear change in the contact barrier between MoS_2_ and graphene. A Schottky barrier and ohmic barrier exist in the off and on states of the MoS_2_ FET with the graphene electrodes. The on/off ratio and electron mobility of the MoS_2_ FET with the graphene electrodes are 10^2^ and 2.5 times higher than those of the MoS_2_ FET with the Au/Ti electrodes, respectively. The Schottky barrier between MoS_2_ and graphene is decreased from 51.5 to 0 meV by the back gate voltage. The implication of these results could be of great importance in better understanding the desirable contact formation for high performance MoS_2_ FETs. This FET may be promising for electronic device applications based on next-generation 2D materials.

## Data Availability

Not applicable.

## Supplementary Materials

The following supporting information can be downloaded at: https://www.mdpi.com/article/10.3390/nano12173038/s1, Figure S1: Schematic illustration of sample preparation process; Figure S2: Electrical properties of chemical-vapor-deposition-grown graphene; Figure S3: Schematic and electrical properties of MoS_2_ field-effect transistor with Au/Ti electrodes.
