# Supplementary material for: Tuning Schottky Barrier of Single-Layer MoS2 Field-Effect Transistors with Graphene Electrodes
Source: Nanomaterials (Basel). 2022 Sep 1;12(17):3038. doi: 10.3390/nano12173038 (PMC9458018; doi:10.3390/nano12173038)
Supplement: Supplementary file 1 [file nanomaterials-12-03038-s001.zip › nanomaterials-1876817-supplementary.pdf]

Supporting information

Tuning Schottky Barrier of Single-Layer MoS_2 Field-Effect Transistors with Graphene Electrodes

A-Rang Jang

Division of Electrical, Electronic and Control Engineering, Kongju National University, Cheonan 31080, Korea; arjang@kongju.ac.kr

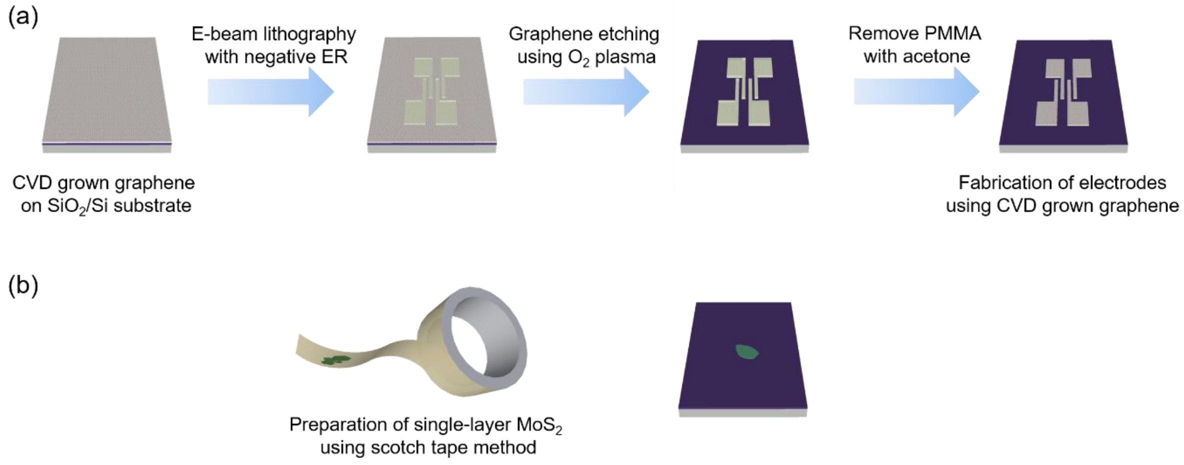

Figure S1. Schematic illustration of preparation process of (a) patterned graphene electrode and (b) preparation of single-layer MoS_2.

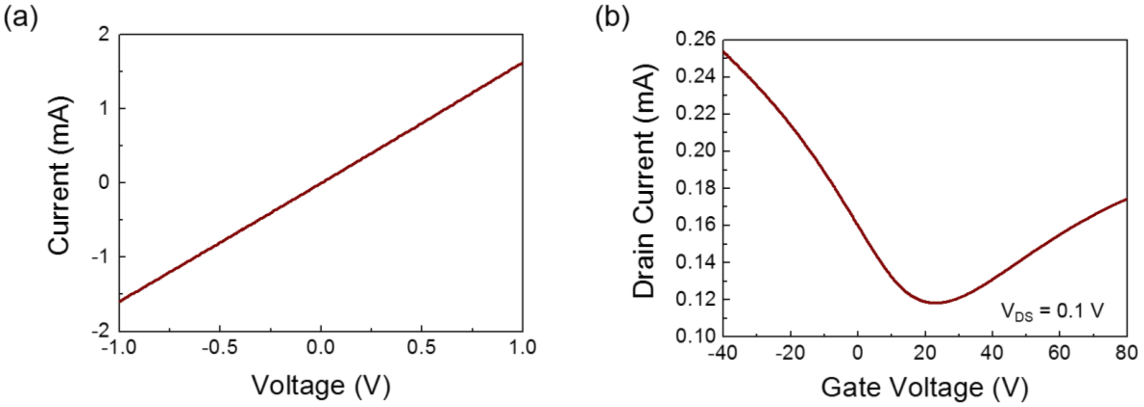

Figure S2. Electrical properties of chemical-vapor-deposition-grown graphene. (a) I_{DS}–V_{DS} output characteristics; (b) I_{DS}–V_g transfer characteristics at V_{DS} = 0.1 V.

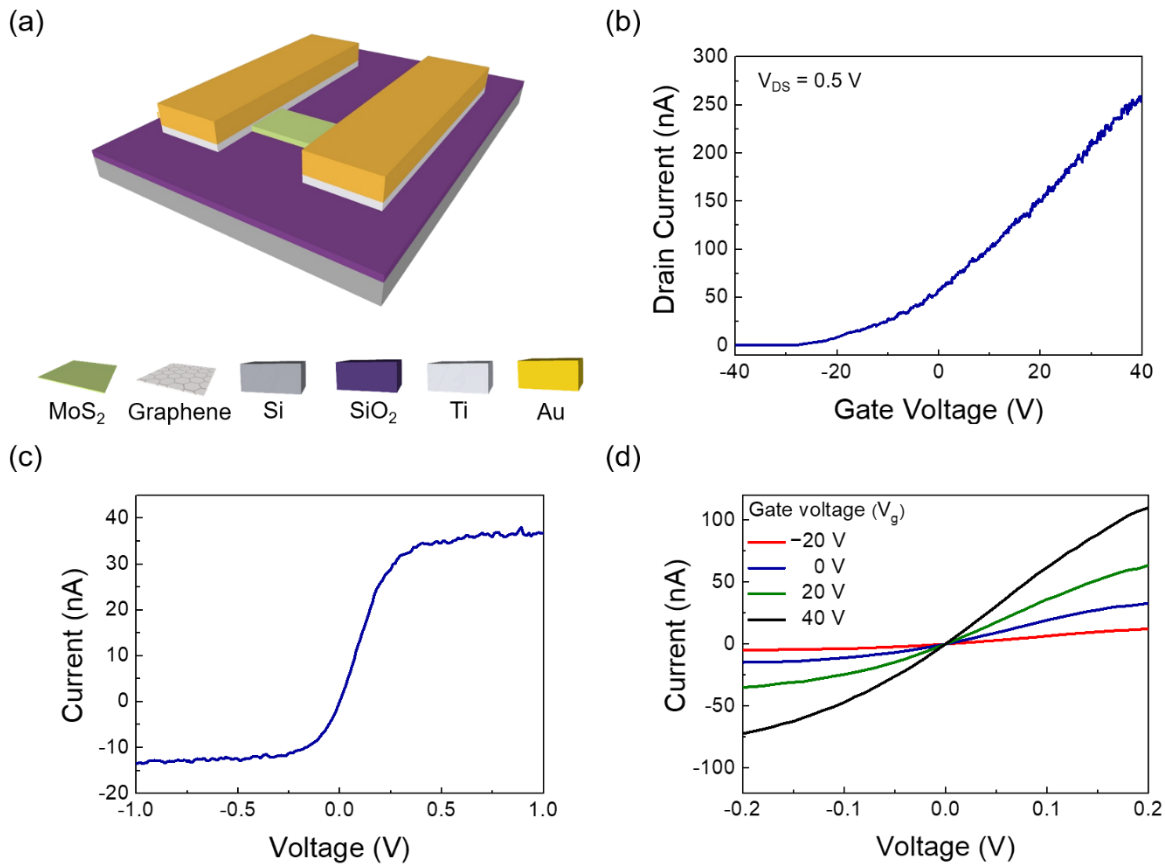

Figure S3. Schematic and electrical properties of MoS_2 field-effect transistor (FET) with Au/Ti electrodes. (a) Schematic of MoS_2 FET with Au/Ti electrodes; (b) I_{DS}–V_g transfer characteristics; (c) I_{DS}–V_{DS} output characteristics; (d) I_{DS}–V_{DS} output characteristics at different gate voltages.
